# Total Versus Inorganic and Organic Species of As, Cr, and Sb in Flavored and Functional Drinking Waters: Analysis and Risk Assessment

**DOI:** 10.3390/molecules25051099

**Published:** 2020-03-01

**Authors:** Wiktor Lorenc, Barbara Markiewicz, Dariusz Kruszka, Piotr Kachlicki, Danuta Barałkiewicz

**Affiliations:** 1 Department of Trace Analysis, Faculty of Chemistry, Adam Mickiewicz University in Poznań, 8 Uniwersytetu Poznańskiego Street, 61-614 Poznań, Poland; wlorenc@amu.edu.pl (W.L.); bpikosz@amu.edu.pl (B.M.); 2 Institute of Plant Genetics, Polish Academy of Sciences, 34 Strzeszyńska street, 60-479 Poznan, Poland; dkru@igr.poznan.pl (D.K.); pkac@igr.poznan.pl (P.K.)

**Keywords:** flavored water, functional water, speciation analysis, risk assessment, ICP-MS, ESI-MS

## Abstract

Packing material can release certain elements such as As, Cr, or Sb into its content and, thus, contaminate the drinking water. The effect of As, Cr, and Sb on human health depends highly on the chemical species in which these elements are introduced into the body. For the above reasons quantification and speciation of As, Cr, and Sb in flavored and functional drinking water samples is an important issue. Total, inorganic, and organic species of As, Cr, and Sb including As(III), As(V), Cr(VI), Sb(III), and Sb(V) were studied in flavored and functional drinking waters. Analyses of total As, Cr, and Sb were conducted using inductively coupled plasma mass spectrometry (ICP-MS) according to ISO 17294-2:2016. The speciation analysis of arsenic, chromium, and antimony in bottled flavored and functional drinking waters was conducted with the use of the elemental (HPLC/ICP dynamic reaction cell (DRC) MS) and molecular (electrospray ionization MS/MS) mass spectrometry. Concentrations of total As, Cr, and Sb (µg∙L^−1^) in waters studied were in the ranges 0.0922 ± 0.0067 to 8.37 ± 0.52, 0.0474 ± 0.0014 to 1.310 ± 0.045, and 0.0797 ± 0.0026 to 1.145 ± 0.019, respectively. Speciation analysis showed that, apart from the toxic ionic species, known and unknown organic species were present in test samples. The risk assessment results proved that there is no risk associated with consumption of these tested beverages in terms of the non-carcinogenic effect of total and inorganic or organic species of As, Cr, and Sb.

## 1. Introduction

Water is a basic nutrient for proper functioning of the human body. Apart from tap water, the main source of water in the diet is bottled water [1,2]. Flavored and functional water-based drinks are currently often recognized as a substitute for drinking water and they can be consumed in volume close to the recommended daily water consumption [3,4,5]. It is known that packing material can release certain elements such as As, Cr, or Sb into bottled water [6,7]. In the literature, one can find reports about the release of elements from various container materials such as polyethylene (PET), glass, aluminum, or steel into beverages such as mineral water, juice, or soft drinks [8,9,10]. It was reported that some water additives such as citric acid can affect both the amount and the species in which certain elements are leached from bottle material [11,12,13,14,15]. In previous work, authors assessed the presence of toxic inorganic As, Cr, and Sb species in mineral water samples proving that those inorganic ionic species were present in that type of water [9,16]. Considering that flavored and functional drinking waters have a rich matrix in comparison to mineral water, they may also contain variable species of As, Cr, and Sb in complexed or other molecular structures. The arsenic and antimony trivalent species (both inorganic and organic) are considered much more toxic than pentavalent species. Inorganic As, Sb, and Cr are all considered as carcinogens to human, although, in the case of chromium, this applies strictly to Cr(VI). Simple organic compounds of As and Sb exhibit lower toxicity than inorganic species, while some species are considered without effect, e.g., arsenobetaine, and the effects decrease even more for complex organic As and Sb compounds such as As- or Sb-sugars [17,18,19,20,21]. Because of the potential risk for consumers, knowledge regarding to contents and chemical species of As, Cr, and Sb as noxious contaminants in flavored and functional drinking waters available from retail outlets is important.

The main goal of the present paper was to the study the presence of As, Cr, and Sb in flavored and functional drinking waters. The authors determined content of total As (TAs), total Cr (TCr), and total Sb (TSb) including speciation analysis of known and unknown As, Cr, and Sb species. An effort was made to identify unknown species of As, Cr, and Sb by using the advanced, sensitive, and accurate techniques such as high-performance liquid chromatography coupled to inductively coupled plasma mass spectrometry (HPLC/ICP-MS) and electrospray ionization mass spectrometry (ESI-MS/MS). The risk assessment of TAs, TCr, and TSb, as well as their inorganic species, potentially arising for consumers of bottled flavored and functional waters was carried out using methods propose by the United States Environmental Protection Agency (USEPA). The risk assessment was conducted for TAs, TCr, and TSb concentrations versus the elemental species concentrations due to the evident differences in toxicity.

## 2. Results

A total number of 42 samples of bottled functional and flavored drinking waters were analyzed. Water with assigned identifier (ID) K.1, despite being labeled as functional drink of the same brand as some other waters, did not contain any functional additives; hence, it was treated as a flavored water. If the concentration was below limit of detection (LOD) values, the result was not included in any further calculations.

### 2.1. The Amounts of TAs, TCr, and TSb

Total As concentration was found above the LOD (0.038 µg∙L^−1^) value in all studied waters. The highest TAs concentration was equal to (8.37 ± 0.52) µg∙L^−1^ and the lowest was (0.0922 ± 0.0067) µg∙L^−1^. Mean As concentration in flavored and functional water samples was equal to 1.606 µg∙L^−1^ and 0.485 µg∙L^−1^, respectively. In the case of chromium, one of the tested samples (G.6) contained TCr at a concentration below the LOD (0.045 µg∙L^−1^) value. The highest chromium concentration was (1.310 ± 0.045) µg∙L^−1^ and the lowest TCr concentration was (0.0474 ± 0.0014) µg∙L^−1^. Mean TCr concentration in flavored and functional water samples was equal to 0.395 µg∙L^−1^ and 0.511 µg∙L^−1^, respectively. For the total antimony quantification in one of the tested samples (I.2), the measured TSb amount was below the LOD (0.061 µg∙L^−1^) value. The highest Sb concentration was (1.145 ± 0.019) µg∙L^−1^ and the lowest Sb concentration was (0.1976 ± 0.0083) µg∙L^−1^. Mean Sb concentration in flavored and functional water samples was equal to 0.372 µg∙L^−1^ and 0.392 µg∙L^−1^, respectively. A total of eight mineral water samples were bought along with the tested samples; these samples are marked with an asterisk in Table S1 (Supplementary Materials) and can be treated as reference matrix samples. Summarized results for amounts of TAs, TCr, and TSb in water samples, including minimal (Min.) and maximal (Max.) values, mean, median, first quartile (Q1), third quartile (Q3), range (R), and coefficient of variation (CV), are collected in Table 1. Detailed results for TAs, TCr, and TSb in water are shown in Table S2 (Supplementary Materials).

### 2.2. The Speciation Analysis of Five Toxic Species of As, Cr, and Sb

Among the two inorganic arsenic species, As(V) was found to be dominant in flavored waters, although, in functional waters, higher concentrations were observed for As(III) in the majority of the tested samples. Highest concentration for As(III) was (0.580 ± 0.055) µg∙L^−1^ and the lowest As(III) concentration was (0.133 ± 0.012) µg∙L^−1^. Mean As(III) concentration was equal to 0.292 µg∙L^−1^ and 0.349 µg∙L^−1^ in flavored and functional water, respectively. Highest concentration for As(V) was (3.26 ± 0.30) µg∙L^−1^ and the lowest As(V) concentration was (0.0600 ± 0.0055) µg∙L^−1^. Mean As(V) concentration was equal to 0.904 µg∙L^−1^ and 0.218 µg∙L^−1^ in flavored and functional water, respectively. In the case of antimony, only Sb(V) was detected from the two analyzed Sb species in one flavored water sample (A.2) at a concentration of (0.524 ± 0.032) µg∙L^−1^. In the case of chromium, Cr(VI) was not detected in any of the tested samples, although, in a number of samples, additional peaks for chromium were detected, one of which was identified as Cr(III), although it was not quantified.

As for the speciation analysis of arsenic beyond previously detected As(V) and As(III), arsenobetaine (AsB) was detected in a number of tested samples. Neither dimethylarsenic acid (DMA) nor monomethylarsenic acid (MMA) were detected in any of the tested samples. Highest AsB concentration was (0.849 ± 0.083) µg∙L^−1^ and the lowest AsB concentration was (0.0541 ± 0.0053) µg∙L^−1^. Mean AsB concentration was equal to 0.312 µg∙L^−1^ and 0.164 µg∙L^−1^ in flavored and functional water, respectively. Agreement of results for As(V) and As(III) obtained with multi-elemental speciation analysis and speciation analysis of arsenic for each sample was tested using Student’s *t*-test; correlation plots were constructed and a coefficient of the determination (*R^2^*) value was estimated. All calculated *t*-values were higher than the critical *t*-value. *R^2^* values for As(III) and As(V) were 0.9894 and 0.9974, respectively.

For about one-half of test samples, the content of toxic arsenic species As(III) and As(V) was equal to about 80% of the total content, while the other about 20% involved AsB and unknown organic species. In the remaining samples, toxic arsenic species As(III) and As(V) corresponded to about 34% to about 75% of TAs, while the remaining percentage constituted AsB and unknown organic species. As for the antimony, in only one sample, SbV corresponded to 100% of TSb content; in all other samples in which TSb was detected, it was present as unknown organic species. In the case of chromium, no Cr(VI) was detected in any sample and, thus, chromium was present as either Cr(III) or other unknown organic Cr species.

**Figure 1:**
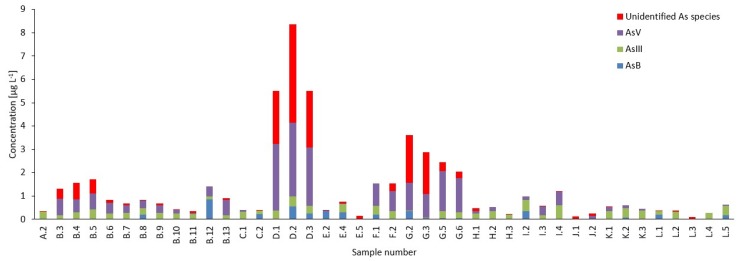
Mass balance for total arsenic (bar height represents total arsenic concentration) and arsenic species in flavored and functional bottled drinking water samples. Unidentified As refers to the difference between total As and sum of As species. Letters in the sample codes denote the same sample brand.

Summarized results for speciation analysis of As, Cr, and Sb in water samples are collected in Table 1. Detailed results for speciation analysis are collected in Table S3 (Supplementary Materials). Mass balance results are shown in Figure 1.

### 2.3. The Screening of Metal Complexes in Water Samples

Based on the results of mass balance, 16 samples were chosen to be subjected to screening of metal complexes in water samples. The selected samples are listed in Table S4 (Supplementary Materials).

In the case of As, in experiments conducted using size exclusion chromatography (SEC), a minimum of one and a maximum of two analytical signals were detected in the range of 30 to 40 min. For antimony, only one analytical signal was detected in the range of 30 to 35 min. In the case of chromium, a minimum of one and a maximum of three analytical signals were detected in the range of 30 to 40 min.

All samples in which signals were detected in the screening of metal complexes in water samples using SEC were subjected to identification of potential organic compounds. A relatively low concentration of As, Cr, and Sb and a high concentration of sample matrix components, especially sugar and citric acid, made it impossible to confirm any compounds containing As, Cr, or Sb. Even though fragmentation spectra were recorded suggesting the presence of organic As compounds, the authors were unable to achieve satisfactory mass error values to fully confirm the presence of these compounds.

Detailed results for screening of metal complexes in water samples are collected in Table S4 (Supplementary Materials).

### 2.4. The Risk Assessment of TAs, TCr, and TSb, and Their Inorganic Species in Bottled Flavored and Functional Drinking Water

Risk assessments were conducted using the estimated daily intake (EDI) estimation and two United States Environmental Protection Agency (USEPA)-recommended methods: target hazard quotient (THQ) and hazard index (HI). Estimation was conducted for TAs, TCr, and TSb concentrations and for the chosen As, Cr, and Sb species: sum of As(III( and As(V) in the case of arsenic, Sb(V) in the case of antimony, and Cr(VI) in the case of chromium.

In the case of total amounts, highest EDI values were estimated for TAs in flavored water samples 0.0069 μg/mg/day and for TCr 0.0022 μg/mg/day in functional water samples when taking into account mean concentrations. In general, EDI values were higher for flavored than functional waters with the exception of Sb in which both values were similar.

The THQ values for As were significantly higher than for Cr and Sb in both flavored and functional bottled drinking water. As for the speciation analysis of flavored and functional bottled drinking water samples, risk assessment could be fully evaluated only in case of arsenic since Sb(V) was only detected in one sample and Cr(VI) was not detected in any of the samples. In the case of As species (sum of As(III) and As(V)), the EDI value was 0.0051 μg/kg/day for flavored water samples and 0.0024 μg/kg/day for functional water samples. EDI for SbV based on one sample was also calculated and was equal to 0.0023 μg/kg/day. THQ values estimated for As exhibited a similar trend to the EDI values and were equal to 0.0010 mg/kg/day and 0.00049 mg/kg/day for flavored and functional bottled drinking water samples, respectively.

Summarized EDI and THQ estimation results are gathered in Table 2. Results of HI estimation for TAs, TCr, and TSb, as well as As and Sb species, in all water types also showed values significantly below 1 mg/kg/day. Results of HI estimation are summarized in Table 3.

## 3. Discussion

A comprehensive speciation analysis consisting of both a quantitative determination of As, Ce, and Sb and an attempt at identification of the species in which they occur in the sample with use of advanced analytical techniques such as (IEC/SEC)/ICP dynamic reaction cell (DRC) MS and complementary ESI-MS/MS were conducted.

All of the three elements of interest were determined in flavored and functional bottled water samples. Arsenic was detected in tested samples with the highest concentration among target elements. Obtained TAs, TCr, and TSb results are in a good accordance with the data available from the literature regarding those element concentrations in various soft drinks and mineral water samples [9,22,23,24,25,26].

Arsenic species were the most prevalent in the analyzed samples. In terms of a bespoke speciation AsB, As(III), and As(V) were detected in flavored and functional bottled drinking water samples; As(V) was found to be a dominant species in flavored water samples, and As(III) was found to be a dominant species in functional water samples. Sb(V) was detected in only one flavored bottled drinking water sample. Cr(VI) was not detected in any of the samples; however, Cr(III) and an unidentified Cr species were detected in several flavored and functional bottled drinking water samples.

As for the non-targeted speciation in (SEC)/ICP-DRC-MS experiments, signals for As, Cr, and Sb were registered, suggesting the presence of different species than those found in bespoke speciation. Following the use of the ESI-MS/MS complimentary technique, fragmentation spectra were recorded, suggesting the presence of organic As compounds, although the authors were not able to fully confirm the presence and structure of those compounds. Antimony was reported in the literature as being complexed by citric acid in soft drinks [11]. In the course of our experiments, we were not able to confirm Sb complexation. There is a lack of data available in the literature regarding organic As, Cr, and Sb in soft drinks.

There is no visible trend in the relationship between TAs, TCr, and TSb or their species presence and concentration in bottled flavored and functional drinking water and the composition of those waters. In around half of the water samples, the known species of As, Cr, and Sb were notably lower than the total element content; these results are different from the results obtained for mineral water samples in our previous experiments [9,16]. Results obtained for known species analysis in flavored and functional bottled drinking water samples are in good accordance with literature data [9,11,20,27,28,29]. The toxic inorganic species of As(III), As(V), Cr(VI), Sb(III), and Sb(V) are important factors that influence the toxicity of these elements in the flavored and functional drinking water samples. When determining only the total content of these elements, their toxicity may be incorrectly estimated.

The value of tolerable daily intake (TDI) was only stated by the World Health Organization (WHO) for As among the analyzed elements and was equal to 15 μg/kg/week (2.1 μg/kg/day); although it was withdrawn without replacement, the authors used this value as a reference [30]. There is no WHO or USEPA reference regarding TDI values for Cr or Sb, although the results obtained for those elements are at a similarly low level to the results for As; thus, the authors can conclude that the risk associated with those elements is similar to the risk associated with As. Calculated EDI values for arsenic target elements and its species were significantly below the TDI value.

The average concentrations of TAs, TCr, TSb, and their inorganic and organic species contained in the flavored and functional drinking water samples were lower than the provisional tolerable weekly intake (PTWI) established by the European Food Safety Authority (EFSA). Moreover, the target hazard quotient (THQ) and the hazard index (HI) were less than one. All values of these parameters were always lower for the determined toxic inorganic species in relation to the total content of elements. For these reasons, it was concluded that the consumption of these two types of beverages does not pose any non-carcinogenic risks and the drinks are safe for consumers. Results obtained during experiments described in this paper were compared with previously obtained results for mineral bottled drinking water samples. In the mineral bottled drinking waters, total As, Cr, and Sb concentrations were in good accordance with the bespoke speciation analysis results; on the contrary, for the flavored and functional bottled drinking water samples, total amounts of As, Cr, and Sb were not in a good accordance with the results from bespoke speciation analysis, suggesting the presence of some different As, Cr, and Sb species [9]. The authors believe that a further investigation into the detailed speciation of toxic elements in various beverages and packing materials is essential in the assessment of the beverage quality and safety.

## 4. Materials and Methods

### 4.1. Sample Collection and Preparation

In total, 42 samples of flavored and functional bottled drinking waters (30 flavored and 12 functional) were obtained. All of the test samples were bought from the same retail outlet in Poland and they were specifically chosen to have a similar expiry date. Samples were grouped based on the brand (marked X.1, X.2, X.3 in Table S1, Supplementary Materials) and they had different flavor and CO_2_ saturation, or contained different additives. Water was stored in original PET bottles in conditions recommended by manufacturer and opened (sampled) directly before instrumental analysis. Before the TAs, TCr, and TSb determination, each individual sample was acidified with high-purity nitric acid to a final acid concentration of 3% and subjected for analysis without pre-filtration or dilution. In the course of all other experiments, samples were analyzed directly without any sample preparation. Detailed characteristics of the samples with their markings are given in Table S1 (Supplementary Materials).

### 4.2. Determination of Total As, Cr, and Sb

Analyses of total As, Cr, and Sb were conducted using ICP-MS according to ISO 17294-2:2016: Water quality—Application of inductively coupled plasma mass spectrometry (ICP-MS)—Part 2: Determination of selected elements including uranium isotopes standard [31]. A dynamic reaction cell (DRC) with oxygen as a reaction gas was employed to remove spectral interferences.

### 4.3. Determination of Inorganic Species of As, Cr, and Sb

We applied the method previously developed in our laboratory for the speciation analysis of five toxic species of As, Cr, and Sb in drinking water samples using IEC/ICP-DRC-MS comprising two speciation procedures [32]. The procedures were extended to ensure that it would apply to flavored and functional bottled drinking waters. Multi-elemental speciation analysis including toxic inorganic species (As(III), As(V), Sb(III), Sb(V), and Cr(VI)) determination was conducted [9,16]. Due to the fact that arsenic was the most abundant element in test samples, additional speciation analysis of arsenic was employed including AsB, As(III), DMA, MMA, and As(V) determination [27]. The authors conducted a chosen parameters validation of the procedure, thereby verifying its applicability in the analysis of flavored and functional bottled drinking waters. A mass balance was conducted based on the difference in total concentrations of the target elements and the results obtained with IEC/ICP-DRC-MS speciation analysis.

### 4.4. Determination of Organic Species of As, Cr, and Sb

The speciation analysis of organic species of As, Cr, and Sb was conducted only in cases when mass balance analysis showed significant differences between amounts of TAs, TCr, and TSb and concentrations of known As, Cr, and Sb species. Screening of metal complexes in water samples using SEC/ICP-DRC-MS was conducted, followed by an identification of possible complexes with the use of ESI-MS/MS [32].

An Elan DRC II ICP spectrometer (PerkinElmer SCIEX, Ontario, Canada), was employed in the course of all ICP-MS experiments. Chromatographic separation was conducted using an HPLC system (PerkinElmer SCIEX, Ontario, Canada) with a PRP-X100 (4.6 mm × 150 mm) (Hamilton, Bonaduz, Switzerland) anion exchange column used for IEC experiments and a Superdex 75 10/300 GL (GE Healthcare, Marlborough, US) size exclusion column used in the course of the SEC experiments. For the identification of potential As, Sb, and Cr organic complexes the high-resolution mass spectrometer Q-Exactive Orbitrap (Thermo Fisher, Bremen, Germany) with a heated electrospray source II (HESI-II) was used. Samples were analyzed with direct injection using a negative ionization mode. Detailed operating conditions for ICP-DRC-MS and IEC/ICP-DRC-MS are summarized in Table S5 (Supplementary Materials), and those for SEC/ICP-DRC-MS and ESI-MS/MS are summarized in Table S6 (Supplementary Materials).

### 4.5. Figures of Merit

For the speciation analysis using IEC/ICP-DRC-MS, chosen validation parameters such as linearity, precision, and trueness were evaluated [33]. Coefficient of correlation (*R*) values were estimated daily for all calibration curves and were greater than 0.99 for all analytes. Evaluation of residual plots showed a random distribution of residuals around the vertical axis. Precision values expressed as coefficient of variation (CV) (%) ranged from 2.7% to 5.9% for multi-elemental speciation analysis and from 3.2% to 5.4% for speciation analysis of arsenic. Trueness was evaluated by applying the standard addition method to water samples and expressed as recovery (%). Recovery values ranged from 92% to 108% for multi-elemental speciation analysis and from 95% to 107% for speciation analysis of arsenic. The measurement uncertainty values, expressed as a percentage of analyte concentration, were as follows: 9.4% for As(III), 9.2% for As(V), 6.1% for Cr(VI), 6.6% for Sb(III), and 6.2% for Sb(V) for multi-elemental speciation analysis, and 9.8% for AsB, 9.9% for As(III), 8.7% for DMA, 9.0% for MMA, and 7.4% for As(V) for speciation analysis of arsenic [32,34]. LOD values were also estimated for the TAs, TCr, and TSb quantification procedure and were as follows: As—0.038 µg∙L^−1^, Cr—0.045 µg∙L^−1^, and Sb—0.061 µg∙L^−1^. Detailed validation results are presented in Table S7 (Supplementary Materials). Measurement traceability was established by applying the standard addition method to water samples.

SEC/ICP-DRC-MS and ESI-MS/MS, as the only qualitative analysis procedures, were not included in the validation process, although each of the samples was analyzed in three replicates in order to roughly assess the repeatability of the experiments.

### 4.6. The Risk Assessment of TAs, TCr, and TSb, and Their Inorganic Species in Bottled Flavored and Functional Drinking Water

The risk assessment was evaluated on the basis of EDI, THQ, and HI. All of the parameters were estimated for TAs, TCr, and TSb amounts and for the chosen As, Cr, and Sb species: sum of As(III) and As(V) in the case of arsenic and Sb(V) in the case of antimony.

EDI was calculated based on the mean and maximal concentrations of TAs, TCr, and TSb, as well as relevant species in bottled flavored and functional drinking water samples. Average soft drink consumption used in the calculations was 94.3 L per capita per year following the UNESDA sales and consumption report for 2017 [35]. Average consumer body weight was fixed at 60 kg. EDI was expressed in (μg/kg/day). The EDI was calculated using the following equation:

EDI = (C × DC)/BW,(1)

where C is the mean/maximal concentration of TAs, TCr, TSb, or relevant species (ug∙L^−1^), DC is the daily bottled flavored or functional drinking water consumption (L∙day^−1^), and BW is the average consumer body weight (kg).

THQ provides the information on the non-carcinogenic risk associated with long-term exposure to a given contaminant and is expressed in mg/kg/day. THQ estimation also includes the reference to a reference dose. Due to the fact that reference doses of As, Cr, and Sb for soft drinks are not stated in EU or WHO standard and guides, reference values for drinking water were employed in the calculations. If the obtained THQ value was <1, the authors concluded that there was no risk associated with the exposure to the contaminant. The THQ was calculated using the following equation:

THQ = (EFr × EDtot × FIR × C)/(RfDo × BW × ATn) × 10^−3^, (2)

where EFr is the frequency of exposure (365 days per year), EDtot is the period of exposure (fixed at 70 years for adults), FIR is the food intake rate (L∙day^−1^), C is the mean/maximal concentration of As, Cr, Sb, or relevant species (∙L^−1^), RfDo is the reference oral dose (As: 10 ug∙L^−1^; Sb: 5 ug∙L^−1^; Cr: 50 ug∙L^−1^) [26], BW is the average consumer body weight (kg), and ATn is the the period of average exposure for non-carcinogens (365 days/year × number of exposure years, 70 years).

HI evaluates the risk assessed with the exposure of one organism to various contaminants. When combining hazard quotients for various contaminants, the toxic mechanism of those contaminants should be the same. An HI value <1 suggests the overall exposure is unlikely to result in non-carcinogenic adverse health effects. HI was calculated as the sum of THQ for TAs, TCr, and TSb or as the sum of THQ for all relevant species [36].

## Figures and Tables

**Table 1:** Summarized results for total amounts of As, Cr, and Sb and speciation analysis results for flavored and functional bottled drinking water samples.

Statistical Parameter	Flavored Bottled Drinking Water					Functional Bottled Drinking Water				
	**As^III^**	**As^V^**	**CrVI**	**Sb^III^**	**Sb^V^**	**As^III^**	**As^V^**	**Cr^VI^**	**Sb^III^**	**Sb^V^**
Min. (µg∙L^−1^)	0.133 ± 0.012	0.0708 ± 0.0065	-	-	0.524 ± 0.032 *	0.158 ± 0.015	0.0600 ± 0.0055	-	-	-
Max. (µg∙L^−1^)	0.414 ± 0.039	3.26 ± 0.30	-	-	0.524 ± 0.032 *	0.580 ± 0.055	0.620 ± 0.057	-	-	-
Mean (µg∙L^−1^)	0.292	0.904	-	-	0.524 ± 0.032 *	0.349	0.218	-	-	-
Median (µg∙L^−1^)	0.291	0.650	-	-	0.524 ± 0.032 *	0.390	0.120	-	-	-
Q1 (µg∙L^−1^)	0.245	0.326	-	-	-	0.250	0.095	-	-	-
Q3 (µg∙L^−1^)	0.339	1.136	-	-	-	0.400	0.267	-	-	-
R (µg∙L^−1^)	0.28	3.19	-	-	-	0.42	0.56	-	-	-
CV (%)	26	97	-	-	-	40	94	-	-	-
	**AsB**	**As^III^**	**DMA**	**MMA**	**As^V^**	**AsB**	**As^III^**	**DMA**	**MMA**	**As^V^**
Min. (µg∙L^−1^)	0.0541 ± 0.0053	0.129 ± 0.013	-	-	0.1103 ± 0.0082	0.0804 ± 0.0079	0.159 ± 0.016	-	-	0.1123 ± 0.0083
Max. (µg∙L^−1^)	0.849 ± 0.083	0.432 ± 0.043	-	-	3.16 ± 0.23	0.350 ± 0.034	0.600 ± 0.059	-	-	0.594 ± 0.044
Mean (µg∙L^−1^)	0.312	0.288	-	-	0.945	0.164	0.350	-	-	0.271
Median (µg∙L^−1^)	0.247	0.292	-	-	0.683	0.174	0.381	-	-	0.161
Q1 (µg∙L^−1^)	0.203	0.249	-	-	0.324	0.080	0.254	-	-	0.115
Q3 (µg∙L^−1^)	0.370	0.353	-	-	1.143	0.200	0.401	-	-	0.372
R (µg∙L^−1^)	0.79	0.30	-	-	3.05	0.27	0.44	-	-	0.48
CV (%)	73	28	-	-	94	78	41	-	-	77.5
	**tAs**		**tCr**	**tSb**		**tAs**		**tCr**	**tSb**	
Min. (µg∙L^−1^)	0.1496 ± 0.0090		0.0740 ± 0.066	0.0797 ± 0.0026		0.0922 ± 0.0067		0.0474 ± 0.0014	0.1976 ± 0.0083	
Max. (µg∙L^−1^)	8.37 ± 0.52		1.252 ± 0.097	1.145 ± 0.019		1.22 ± 0.023		1.310 ± 0.045	0.6125 ± 0.0066	
Mean (µg∙L^−1^)	1.606		0.395	0.372		0.485		0.511	0.392	
Median (µg∙L^−1^)	0.817		0.380	0.275		0.411		0.514	0.431	
Q1 (µg∙L^−1^)	0.432		0.193	0.222		0.258		0.202	0.298	
Q3 (µg∙L^−1^)	1.678		0.465	0.443		0.587		0.690	0.469	
R (µg∙L^−1^)	8.22		1.18	1.07		1.13		1.26	0.41	
CV (%)	117		65	66		67.5		76	32	

* Sb^V^ was detected in only one flavored bottled drinking water sample. Min.—minimum; Max.—maximum; Q1—first quartile; Q3—third quartile; R—range; CV—coefficient of variation.

**Table 2:** Estimated daily intake (EDI) and target hazard quotient (THQ) values for flavored and functional bottled drinking water samples estimated for mean and maximal As, Cr, and Sb, as well as As and Sb species concentrations.

		EDI Mean * (μg/kg/day)	THQ Mean * (mg/kg/day)	EDI Max * (μg/kg/day)	THQ Max * (mg/kg/day)
TAs	Flavored	0.0069	0.0014	0.036	0.0072
	Functional	0.0021	0.00042	0.0053	0.0011
TCr	Flavored	0.0017	0.000034	0.0054	0.00011
	Functional	0.0022	0.000044	0.0056	0.00011
TSb	Flavored	0.0016	0.00016	0.0049	0.00049
	Functional	0.0017	0.00017	0.0026	0.00026
As species **	Flavored	0.0051	0.0010	0.016	0.0032
	Functional	0.0024	0.00049	0.0052	0.0010
Sb ^V^ ***	Flavored	0.0023	0.00023	0.0023	0.00023

* Tolerable daily intake (TDI) stated by World Health Organization (WHO) for As is equal to 2.1 (μg/kg/day), while the limit value for THQ is set at 1 (mg/kg/day). ** Sum of As^III^ and As^V^; *** Sb^V^ was detected in only one flavored bottled drinking water sample.

**Table 3:** Hazard index (HI) values for flavored and functional bottled drinking water samples estimated for mean and maximal As, Cr, and Sb, as well as As and Sb species concentrations.

		HI Mean * (mg/kg/day)	HI Max * (mg/kg/day)
Total As, Cr, and Sb	Flavored	0.0016	0.0078
	Functional	0.00063	0.0014
As, Cr, and Sb species	Flavored	0.0013	0.0034
	Functional	0.00049	0.0010

* Limit value for HI is set at 1 (mg/kg/day).

## Supplementary Materials

The following are available online at https://www.mdpi.com/1420-3049/25/5/1099/s1: Table S1. Sample characteristics; Table S2. Water sample results for total amount of As, Cr, and Sb; Table S3. Water sample results for multi-elemental speciation analysis and speciation analysis of arsenic; Table S4. Water sample results for screening of metal complexes in water samples; Table S5: Operating parameters for HPLC and ICP-DRC-MS for total As, Cr, and Sb determination and speciation analysis; Table S6: Operating parameters for (SEC)/ICP-DRC-MS and ESI-MS/MS; Table S7. Analytical procedure parameters.
