# Supplementary material for: Total Versus Inorganic and Organic Species of As, Cr, and Sb in Flavored and Functional Drinking Waters: Analysis and Risk Assessment
Source: Molecules. 2020 Mar 1;25(5):1099. doi: 10.3390/molecules25051099 (PMC7179223; doi:10.3390/molecules25051099)
Supplement: Supplementary file 1 [file molecules-25-01099-s001.pdf]

Table S1. Samples characteristic

Sample	Flavor	Carbonated/ non-carbonated**	pH	Mineralization [mg L^{-1}]	Bottle Color	Composition stated by manufacturer (with functional additives in bold)
A.1*	Mineral	NC	6.52	1670.9	Colorless	-
A.2	Lemon	NC	5.91	1670.9	Light Blue	Mineral water, natural lemon flavor with other natural flavors
B.1*	Mineral	NC	7.27	230	Light Blue	-
B.2*	Mineral	C	4.84	311.5	Blue	-
B.3	Apple	C	2.89	-	Colorless	Mineral water, sugar, apple juice from concentrate, apple extract, citric acid, natural flavor
B.4	Orange	C	3.03	-	Colorless	Spring water, sugar, orange juice from concentrate, citric acid, locust bean gum, ascorbic acid, carotenoid colorants, potassium sorbate, natural flavors
B.5	Lemon	C	2.75	-	Colorless	Spring water, sugar, lemon juice from concentrate, citric acid, locust bean gum, ascorbic acid, carotenoid colorants, potassium sorbate, natural flavors
B.6	Wild strawberry	NC	3.02	-	Light Blue	Mineral water, sugar, citric acid, natural wild strawberry flavor with other natural flavors
B.7	Apple	NC	3.04	-	Light Blue	Spring water, sugar, citric acid, natural flavor, apple juice from concentrate
B.8	Strawberry	NC	2.89	-	Light Blue	Spring water, sugar, citric acid, natural flavor, strawberry juice from concentrate
B.9	Lemon	NC	2.74	-	Light Blue	Spring water, sugar, citric acid, natural lemon flavor with other natural flavors
B.10	Peach	NC	3.01	-	Light Blue	Spring water, sugar, citric acid, sodium benzoate, natural peach flavor with other natural flavors
B.11	Cherry	NC	2.90	-	Light Blue	Spring water, sugar, citric acid, sodium benzoate, natural cherry flavor with other natural flavors
B.12	Cherry	NC	3.05	-	Colorless	Mineral water, sugar, cherry juice from concentrate, apple juice from concentrate, citric acid, black carrot juice concentrate, natural flavors
B.13	Lemon	NC	2.83	-	Colorless	Mineral water, sugar, lemon juice from concentrate, apple juice from concentrate, citric acid, locust bean

						gum, ascorbic acid, natural lemon flavor with other natural flavors
C.1	Strawberry	NC	2.63	-	Colorless	Water, sugar, apple juice from concentrate, citric acid, strawberry juice from concentrate, natural flavor
C.2	Lemon	NC	2.65	-	Colorless	Water, sugar, lemon juice from concentrate, citric acid, natural flavor, ascorbic acid
D.1	Lemon	NC	3.64	775.2	Yellow	Mineral water, sugar, lemon juice from concentrate, grape juice from concentrate, flavor, citric acid, sodium citrate, ascorbic acid
D.2	Apple	NC	3.42	775.2	Green	Mineral water, sugar, apple juice from concentrate, grape juice from concentrate, flavor, citric acid, ascorbic acid
D.3	Raspberry	NC	3.47	775.2	Red	Mineral water, sugar, raspberry juice from concentrate, grape juice from concentrate, flavor, citric acid, sodium citrate, ascorbic acid
E.1*	Mineral	NC	6.50	1010	Light Blue	-
E.2	Strawberry	NC	2.79	-	Blue	Water, sugar, citric acid, strawberry juice from concentrate, ascorbic acid, flavor
E.3*	Mineral	C	5.53	1010	Light Green	-
E.4	Strawberry	C	3.77	-	Colorless	Mineral water, citric acid, natural strawberry flavor with other natural flavors, sweeteners (saccharin, cyclamate)
E.5	Lemon	C	4.2	-	Colorless	Mineral water, citric acid, natural lemon flavor with other natural flavors, sweeteners (saccharin, cyclamate)
F.1	Strawberry	NC	3.2	-	Colorless	Mineral water, sugar, citric acid, natural strawberry flavor with other natural flavors, sodium benzoate, potassium sorbate, sweeteners (saccharin, cyclamate, acesulfame k, aspartame)
F.2	Lemon	NC	2.99	-	Colorless	Mineral water, sugar, citric acid, natural lemon flavor with other natural flavors, sodium benzoate, potassium sorbate, sweeteners (saccharin, cyclamate, acesulfame k, aspartame)

G.1*	Mineral	C	5.32	646.5	Light Green	-
G.2	Orange and Lemon	C	3.32	-	Light Green	Mineral water, glucose-fructose syrup, sugar, citric acid, natural citric flavor with other natural flavors, natural orange flavor, potassium sorbate, sweeteners (acesulfame k, sucralose)
G.3	Peach and apple	C	3.37	-	Light Green	Mineral water, glucose-fructose syrup, sugar, citric acid, natural peach flavor with other natural flavors, natural apple flavor, potassium sorbate, sweeteners (acesulfame k, sucralose)
G.4*	Mineral	NC	7.05	-	Light Blue	-
G.5	Strawberry	NC	3.47	-	Light Blue	Mineral water, glucose-fructose syrup, sugar, citric acid, natural strawberry flavor with other natural flavors, sweeteners (acesulfame k, sucralose)
G.6	Lemon	NC	3.26	-	Light Blue	Mineral water, glucose-fructose syrup, sugar, citric acid, natural lemon flavor with other natural flavors, natural grapefruit flavor, sweeteners (acesulfame k, sucralose)
H.1	Lemon	NC	3.08	-	Colorless	Water, sugar, lemon juice from concentrate, flavor
H.2	Blueberry	NC	3.14	-	Colorless	Water, sugar, lemon juice from concentrate, blueberry juice from concentrate, flavor
H.3	Raspberry	NC	3.12	-	Colorless	Water, sugar, lemon juice from concentrate, raspberry juice from concentrate, flavor
I.1*	Mineral	NC	7.80	387.4	Colorless	-
I.2	Red grape and dragon fruit	NC	2.70	-	Colorless	Water, sugar, citric acid, black carrot juice concentrate, natural grape flavor with other natural flavors, **niacin, vit. B6, biotin, vit. B12, zinc lactate**
I.3	Mint, dandelion and nettle	NC	3.10	-	Colorless	Water, fructose, citric acid, natural flavor, tarragon extract, mint extract, ascorbic acid, dandelion root extract, nettle root extract, **niacin, pantothenic acid, vit. B6, folic acid, biotin, vit. B12**
I.4	Lemon and orange	NC	3.70	-	Blue	Water, sugar, citric acid, natural flavors, niacin, **pantothenic acid, vit. B6, folic acid, biotin, vit. B12, magnesium carbonate,** ascorbic acid

J.1	Fruit flavor (non-specified)	NC	4.07	-	Colorless	Water, citric acid, sodium citrate, **vit. C, niacin, vit. B6, vit. B12, pantothenic acid, zinc,** cyclamates, saccharin, aspartame, acesulfame K, potassium sorbate, sodium benzoate, gum Arabic, esters of glycerol and plant resin, flavors, carotenes
J.2	Fruit flavor (non-specified)	NC	3.90	-	Colorless	Water, citric acid, sodium citrate, concentrated aronia juice, **vit. C, niacin, vit. B6, vit. B12, pantothenic acid, caffeine, guarana extract**, cyclamates, saccharin, aspartame, acesulfame K, potassium sorbate, sodium benzoate, flavors, ammonia-sulphite caramel
K.1	Raspberry	NC	2.98	-	Colorless	Water, sugar, raspberry juice from concentrate, lemon juice from concentrate, flavor
K.2	Blueberry	NC	2.97	-	Colorless	Water, cane sugar, blueberry juice from concentrate, lemon juice from concentrate, **niacin, vit. B6, folic acid, vit. B12, biotin, pantothenic acid**, flavors
K.3	Citrus mix	NC	2.98	-	Colorless	Water, cane sugar, lemon and grapefruit juice from concentrate, **niacin, vit. B6, folic acid, vit. B12, biotin, pantothenic acid,** flavors
L.1	Grapefruit	NC	3.92	-	Light Blue	Water, sugar, glucose-fructose syrup, citric acid, sodium citrate, natural grapefruit flavor, **magnesium citrate,** ascorbic acid, sodium benzoate
L.2	Kiwi	NC	3.37	-	Light Blue	Natural mineral water, sugar, glucose-fructose syrup, citric acid, sodium citrate, natural kiwi flavor, flavor, ascorbic acid, **caffeine, niacin,** sodium benzoate
L.3	Pineapple	NC	3.38	-	Light Blue	Mineral water, citric acid, sweeteners (acesulfame k, sucralose), natural pineapple flavor, flavor, **zinc sulfate**, sodium benzoate
L.4	Mandarin	NC	3.24	-	Light Blue	Mineral water, sugar, glucose-fructose syrup, citric acid, sodium citrate, ascorbic acid, natural mandarin flavor, **niacin, folic acid, vit. B12**, sodium benzoate
L.5	Rhubarb and green coffee	NC	3.41	-	Light Blue	Water, sugar, glucose-fructose syrup, citric acid, sodium citrate, rhubarb juice from concentrate, **guarana extract, green coffee extract**, ascorbic acid, sodium benzoate, **folic acid, niacin**

*- Mineral/non-functional bottled drinking water sample
**C — carbonated water, NC — non-carbonated water

Table S2. ~~Real~~ Water samples result for total amount of As, Cr and Sb

Sample	Total concentration (C±SD) [µg L^{-1}]		
	As	Cr	Sb
A.1	0.0078±0.0.0011	0.0115±0.0014	0.1857±0.0059
A.2	0.318±0.020	0.0740±0.0066	0.503±0.015
B.1	0.184±0.012	0.0473±0.0034	0.2568±0.0098
B.2	0.587±0.052	0.0520±0.0044	0.3760±0.0094
B.3	1.320±0.069	0.847±0.023	0.1825±0.0035
B.4	1.575±0.070	0.581±0.014	0.846±0.027
B.5	1.713±0.063	1.252±0.097	0.748±0.031
B.6	0.825±0.026	0.2100±0.0091	0.1786±0.0051
B.7	0.681±0.032	0.1742±0.0074	0.2855±0.0079
B.8	0.809±0.023	0.193±0.015	0.264±0.015
B.9	0.689±0.035	0.1882±0.0056	0.2874±0.0062
B.10	0.416±0.024	0.2724±0.0059	0.3029±0.0074
B.11	0.360±0.017	0.371±0.024	0.332±0.017
B.12	1.380±0.053	0.924±0.020	0.1974±0.0029
B.13	0.895±0.069	0.408±0.026	0.2269±0.0049
C.1	0.364±0.024	0.4465±0.0090	1.145±0.019
C.2	0.405±0.026	0.1665±0.0084	0.787±0.030
D.1	5.52±0.24	0.502±0.020	0.458±0.010
D.2	8.37±0.52	0.525±0.027	0.643±0.037
D.3	5.50±0.23	0.439±0.024	0.598±0.019
E.1	0.0528±0.0074	0.0415±0.0029	0.1545±0.0048
E.2	0.386±0.018	0.3993±0.0061	0.0797±0.0026
E.3	0.251±0.013	0.0427±0.0027	0.2545±0.0034
E.4	0.754±0.018	0.392±0.023	0.2262±0.0054
E.5	0.1496±0.0090	0.204±0.012	0.1884±0.0034
F.1	1.504±0.040	0.4848±0.0090	0.2278±0.0063
F.2	1.536±0.086	0.465±0.032	0.260±0.010
G.1	1.077±0.072	0.0150±0.0012	0.5099±0.0056
G.2	3.60±0.29	0.3801±0.0034	0.2631±0.0033
G.3	2.875±0.095	0.4099±0.0046	0.1915±0.0032

G.4	0.933±0.014	0.3225±0.0012	0.2541±0.0019
G.5	2.446±0.096	0.1785±0.0015	0.2287±0.0012
G.6	2.038±0.059	0.0220±0.0069*	0.1212±0.0021
H.1	0.480±0.018	0.1929±0.0035	0.3873±0.0073
H.2	0.520±0.013	0.1897±0.0086	0.378±0.017
H.3	0.221±0.022	0.234±0.012	0.395±0.10
I.1	0.490±0.020	0.00206±0.00019	0.156±0.0075
I.2	0.922±0.012	0.940±0.041	0.001079±0.000015*
I.3	0.584±0.027	0.570±0.029	0.2701±0.0072
I.4	1.220±0.023	1.310±0.045	0.6125±0.0066
J.1	0.1259±0.0035	0.538±0.023	0.489±0.010
j.2	0.240±0.014	0.490±0.015	0.469±0.012
K.1	0.546±0.038	0.3466±0.0090	0.2202±0.0052
k.2	0.578±0.019	0.904±0.018	0.2233±0.0044
K.3	0.445±0.019	0.619±0.034	0.1976±0.0083
L.1	0.376±0.012	0.217±0.012	0.326±0.011
L.2	0.377±0.014	0.0759±0.0068	0.437±0.015
L.3	0.0922±0.0067	0.1581±0.0045	0.3808±0.0058
L.4	0.2643±0.0066	0.0474±0.0014	0.4691±0.0069
L.5	0.594±0.022	0.2584±0.0029	0.4314±0.0067
* - results below LOD; LOD values: As – 0.038 µg L^{-1}, Cr – 0.045 µg L^{-1}, Sb – 0.061 µg L^{-1}			

Table S3. ~~Real~~ Water samples result for multielemental speciation analysis and speciation analysis of arsenic

Sample	Multielemental speciation analysis (C±U) [µg L^{-1}]							Speciation analysis of arsenic (C±U) [µg L^{-1}]				
	As^{III}	As^{V}	Cr^{VI}	Cr^{III}	Cr?	Sb^{III}	Sb^{V}	AsB	As^{III}	DMA	MMA	As^{V}
A.1	-	-	-	-	-	-	0.201±0.012	-	-	-	-	-
A.2	0.327±0.031	-	-	+	+	-	0.524±0.032	-	0.317±0.031	-	-	-
B.1	-	0.175±0.016	-	-	-	-	0.234±0.014	-	-	-	-	0.165±0.012
B.2	-	0.574±0.053	-	-	-	-	0.300±0.019	-	-	-	-	0.565±0.042
B.3	0.194±0.018	0.701±0.064	-	+	+	-	-	-	0.183±0.018	-	-	0.688±0.051
B.4	0.310±0.029	0.570±0.052	-	+	+	-	-	-	0.306±0.030	-	-	0.555±0.041
B.5	0.400±0.038	0.650±0.060	-	+	+	-	-	-	0.421±0.042	-	-	0.690±0.051
B.6	0.269±0.025	0.460±0.042	-	+	+	-	-	-	0.252±0.025	-	-	0.451±0.033
B.7	0.284±0.027	0.326±0.030	-	+	+	-	-	-	0.281±0.028	-	-	0.311±0.023
B.8	0.297±0.028	0.340±0.031	-	+	+	-	-	0.194±0.019	0.276±0.027	-	-	0.328±0.024
B.9	0.283±0.027	0.327±0.030	-	+	+	-	-	-	0.274±0.027	-	-	0.322±0.024
B.10	0.279±0.026	0.140±0.013	-	+	+	-	-	-	0.260±0.025	-	-	0.148±0.011
B.11	0.233±0.022	0.0501±0.0046*	-	+	+	-	-	-	0.231±0.023	-	-	0.0449±0.0033*
B.12	0.133±0.012	0.453±0.042	-	+	+	-	-	0.849±0.083	0.129±0.013	-	-	0.434±0.032
B.13	0.175±0.016	0.654±0.060	-	+	+	-	-	-	0.164±0.016	-	-	0.679±0.050
C.1	0.332±0.031	0.0708±0.0065	-	-	-	-	-	-	0.329±0.033	-	-	0.0714±0.0053*
C.2	0.166±0.016	-	-	+	+	-	-	0.230±0.023	0.155±0.015	-	-	-
D.1	0.397±0.037	2.72±0.25	-	+	+	-	-	-	0.379±0.038	-	-	2.86±0.21
D.2	0.414±0.039	3.26±0.30	-	+	+	-	-	0.554±0.054	0.432±0.043	-	-	3.16±0.23
D.3	0.342±0.032	2.41±0.22	-	+	+	-	-	0.247±0.024	0.321±0.032	-	-	2.52±0.19
E.1	-	0.049±0.0046*	-	-	+	-	0.1046±0.0065	-	-	-	-	0.0510±0.0038*
E.2	-	-	-	+	+	-	-	0.381±0.037	-	-	-	-
E.3	-	-	-	-	-	-	0.1512±0.0094	0.183±0.018	-	-	-	-
E.4	0.331±0.031	0.0293±0.0027*	-	-	-	-	-	0.298±0.029	0.354±0.035	-	-	0.0293±0.0022*

E.5	-	-	-	+	+	-	-	-	-	-	-	-
F.1	0.380±0.036	0.994±0.091	-	-	-	-	-	0.212±0.021	0.364±0.036	-	-	0.955±0.071
F.2	0.333±0.031	0.824±0.076	-	-	-	-	-	-	0.350±0.035	-	-	0.851±0.063
G.1	-	0.991±0.091	-	-	-	-	0.433±0.027	-	-	-	-	1.023±0.076
G.2	0.0109±0.0010 *	1.23±0.11	-	-	-	-	-	0.358±0.035	0.0102±0.0010 *	-	-	1.191±0.088
G.3	0.0342±0.0032 *	1.041±0.096	-	-	-	-	-	0.0442±0.0043*	0.0327±0.0032 *	-	-	1.000±0.074
G.4	-	0.900±0.083	-	-	-	-	0.0362±0.0022 *	-	-	-	-	0.957±0.071
G.5	0.286±0.027	1.68±0.15	-	+	+	-	-	0.0541±0.0053	0.302±0.030	-	-	1.72±0.13
G.6	0.241±0.023	1.51±0.14	-	+	+	-	-	0.0546±0.0054	0.249±0.025	-	-	1.47±0.11
H.1	0.259±0.024	0.117±0.011	-	+	+	-	-	-	0.249±0.025	-	-	0.1103±0.0082
H.2	0.343±0.032	0.168±0.015	-	+	+	-	-	-	0.360±0.036	-	-	0.164±0.012
H.3	0.204±0.019	-	-	+	+	-	-	-	0.199±0.020	-	-	-
I.1	0.175±0.016	0.310±0.029	-	-	-	-	0.1240±0.0077	-	0.180±0.018	-	-	0.299±0.022
I.2	0.490±0.046	0.165±0.015	-	+	+	-	-	0.350±0.034	0.471±0.047	-	-	0.161±0.012
I.3	0.158±0.015	0.370±0.034	-	-	-	-	-	0.00506±0.00050*	0.166±0.016	-	-	0.372±0.027
I.4	0.580±0.055	0.620±0.057	-	+	+	-	-	-	0.600±0.059	-	-	0.594±0.044
J.1	-	-	-	+	+	-	-	-	-	-	-	-
J.2	0.0300±0.0028 *	0.110±00.010	-	-	-	-	-	-	0.0280±0.0028 *	-	-	0.1123±0.0083
K.1	0.370±0.035	0.169±0.016	-	-	-	-	-	-	0.357±0.035	-	-	0.173±0.013
K.2	0.400±0.038	0.120±0.011	-	+	+	-	-	0.0804±0.0079	0.401±0.04	-	-	0.1145±0.0085
K.3	0.390±0.037	0.0800±0.0074	-	+	+	-	-	-	0.381±0.038	-	-	0.0798±0.0059 *
L.1	0.170±0.016	-	-	-	-	-	-	0.200±0.020	0.159±0.016	-	-	-
L.2	0.310±0.029	-	-	-	-	-	-	-	0.319±0.032	-	-	-
L.3	-	-	-	-	-	-	-	-	-	-	-	-
L.4	0.250±0.024	-	-	-	-	-	-	0.0151±0.0015*	0.254±0.025	-	-	-
L.5	0.390±0.037	0.0600±0.0055	-	-	-	-	-	0.174±0.017	0.401±0.040	-	-	0.0612±0.0045 *

* Results below LOD, +/- Detected/Undetected

Table S4. ~~Real~~ Water samples result for screening of metal complexes in water samples

Sample	(number of peaks)<br>retention times		
	As	Cr	Sb
B.3	Nd.	(1)<br>RT_1 = 31.5 min.	(1)<br>RT_1 = 31.5 min.
B.4	(1)<br>RT_1 = 34 min.	(2)<br>RT_1 = 30.5 min.<br>RT_2 = 33.5 min.	(1)<br>RT_1 = 31.5 min.
B.5	(1)<br>RT_1 = 34 min.	(3)<br>RT_1 = 30.5 min.<br>RT_2 = 34 min.<br>RT_3 = 36 min.	(1)<br>RT_1 = 31.5 min.
B.12	(1)<br>RT_1 = 33.8 min.	(1)<br>RT_1 = 30.5 min.	(1)<br>RT_1 = 31.5 min.
C.1	Nd.	(2)<br>RT_1 = 30.5 min.<br>RT_2 = 34 min.	(1)<br>RT_1 = 31.5 min.
D.1	(2)<br>RT_1 = 31.5 min.<br>RT_2 = 33 min.	Nd.	(1)<br>RT_1 = 31.5 min
D.2	(1)<br>RT_1=32 min	Nd.	(1)<br>RT_1 = 31.5 min
D.3	(1)<br>RT_1=32 min	Nd.	(1)<br>RT_1 = 31.5 min
F.1	Nd.	(3)<br>RT_1 = 30.5 min.	(1)<br>RT_1 = 31.5 min.

		RT_2 = 34 min.<br>RT_3 = 36 min.	
F.2	Nd.	(3)<br>RT_1 = 30.5 min.<br>RT_2 = 34 min.<br>RT_3 = 36 min.	(1)<br>RT_1 = 31.5 min.
G.2	(2)<br>RT_1 = 31 min.<br>RT_2 = 33 min.	(3)<br>RT_1 = 30.5 min.<br>RT_2 = 34 min.<br>RT_3 = 36 min.	(1)<br>RT_1 = 31.5 min.
G.3	(1)<br>RT_1=31 min	(3)<br>RT_1 = 30.5 min.<br>RT_2 = 34 min.<br>RT_3 = 36 min.	(1)<br>RT_1 = 31.5 min.
G.5	(2)<br>RT_1 = 32 min.<br>RT_2 = 34 min.	(3)<br>RT_1 = 30.5 min.<br>RT_2 = 34 min.<br>RT_3 = 36 min.	(1)<br>RT_1 = 31 min.
G.6	(1)<br>RT_1 = 31.5 min.	(1)<br>RT_1 = 30.5 min.	(1)<br>RT_1 = 31 min.
I.4	(1)<br>RT_1 = 33 min.	(1)<br>RT_1 = 30.5 min.	(1)<br>RT_1 = 31 min.
K.2	(1)<br>RT_1=31 min	(2)<br>RT_1 = 30.5 min.<br>RT_2 = 36 min.	(1)<br>RT_1=31 min

Table S5: Operating parameters for HPLC and ICP-DRC-MS for total As, Cr and Sb determination and speciation analysis

Parameter	Setting	
	ICP-MS	
Instrument	PE Sciex ELAN 6100 DRC II	
RF Power	1050-1200 W	
Nebulizer gas (Ar) flow rate	0.89-0.93 L min^{-1}	
Auxilary gas (Ar) flow rate	1.20 L min^{-1}	
Plasma gas (Ar) flow rate	16 L min^{-1}	
Sampler and skimmer cones	Pt	
Lens voltage	6.5-10.0 V	
Detector mode	Dual	
Data collection mode	^{91}AsO, ^{52}Cr, ^{121}Sb	
Internal standard*	^{74}Ge for ^{91}AsO, ^{103}Rh for ^{52}Cr and ^{121}Sb	
Scan mode	Peak hopping	
DRC gas (O_2) flow rate	0.55 L min^{-1}	
Rpq	0.55	
Rpa	0	
	HPLC	
	Multielemental speciation analysis	Speciation analysis of arsenic
Instrument	PE series 200 HPLC pump<br>PE series 225 HPLC autosampler<br>PE series 200 column oven	
Column	Hamilton PRP-X100	
Elution	Gradient**	Isocratic
Mobile Phase	3mM $EDTANa_2$<br>36mM NH_4NO_3<br>pH: Eluent A = 4.6, Eluent B = 9.0	10 mM $NH_4H_2PO_4$<br>10 mM NH_4NO_3<br>pH = 9.2
Mobile phase flow rate	1.2 mL min^{-1}	
Sample injection volume	100 µL	
Column temperature	25ºC	

*For total amounts determination only.

**Step 1 (equilibration) – 0.5 minute of 100% eluent A, step 2 (run) – 0.1 minute of 100% eluent A, step 3 (run) – 0.1 minute of skipping from 100% eluent A to 100% eluent B, step 4 (run) – 4.3 minute of 100% eluent B, step 5 (run) – 0.1 minute of skipping from 100% eluent B to 100% eluent A, step 6 (run) – 2.9 minutes of 100% eluent A, step 7 (wash) – 6.9 minutes of 100% eluent A.

Table S6: Operating parameters for (SEC)/ICP-DRC-MS and ESI-MS/MS

Parameter	Setting
	(SEC)/ICP-DRC-MS
Instrument	PE series 200 HPLC pump<br>PE series 225 HPLC autosampler<br>PE series 200 column oven<br>PE series 200 UV/Vis detector
Column	Superdex 75 10/300 GL SEC
Elution	Isocratic
Mobile Phase	50 mM $NH_4H_2PO_4$,<br>30 mM NaCl<br>pH: 7.2
Mobile phase flow rate	0.55 mL min^{-1}
Sample injection volume	100 µL
Column temperature	25ºC
	ESI-MS/MS
Instrument	Q-Exactive Orbitrap MS with a heated electrospray source II (HESI-II)
Syringe pump flow rate	5 µl·min^{-1}
Ion source	HESI -II
FullMS-ddMS2 range	100-1000 *m/z*
FullMS resolution	70 000
ddMS2 resolution	17 500
Sheath gas (N_2) flow rate	15 units
Auxilary gas (N_2) flow rate	5 units
Auxilary gas temperature	120 °C
Electrospray Voltage	-2.5 kV
Ion transfer tube temperature	250 °C
S-lens RF	50
Collision energy	30 eV

Table S7. Analytical procedures parameters

Analytical Procedure Parameter	Analyte				
Multielemental speciation analysis	As^{III}	As^{V}	Cr^{VI}	Sb^{III}	Sb^{V}
Retention Time [min]	1.3	2.1	7.1	4.5	1.7
Linear range* [µg L^{-1}]	0.2-5.0	0.2-5.0	0.5-5.0	0.5-5.0	0.1-5.0
Coefficient of determination	0.9952-0.9999	0.9974-1.0000	0.9986-0.9999	0.9991-0.9999	0.9990-1.0000
LOD* [µg L^{-1}]	0.058	0.051	0.12	0.090	0.046
Recovery (at 0.5 µg L^{-1}) [%]	95	103	92	99	108
Intermediate Precision [%]	3.3	2.7	4.3	5.9	4.2
Expanded Uncertainty* (k=2) [% of analyte concentration]	9.4	9.2	6.1	6.6	6.2
Speciation analysis of arsenic	AsB	As^{III}	DMA	MMA	As^{V}
Retention Time [min]	1.5	1.7	2.1	4.1	5.7
Linear range* [µg L^{-1}]	0.5-10.0	0.5-10.0	0.5-10.0	0.5-10.0	0.5-10.0
Coefficient of determination	0.9991-0.9999	0.9989-0.9999	0.9990-0.9999	0.9992-0.9999	0.9993-0.9999
LOD* [µg L^{-1}]	0.054	0.081	0.053	0.10	0.080
Recovery (at 1 µg L^{-1}) [%]	95	102	99	107	99

Intermediate Precision [%]	3.2	4.8	3.2	3.6	5.4
Expanded Uncertainty* (k=2) [% of analyte concentration]	9.8	9.9	8.7	9.0	7.4
Screening of metal complexes in water samples	Conalbumin<br>75kDa	Ovalbumin<br>43kDa	Carbonic anhydrase<br>29kDa	Ribonuclease A<br>13,7kDa	Aprotinin<br>6,5kDa
Retention Time [min]	17.2	18.8	21.5	25.0	35.0

*- Parameters from full validation conducted during earlier experiment and described in our previous paper (Lorenc et al., Molecules 24, 2019).
